# Heavy Metal Stress, Signaling, and Tolerance Due to Plant-Associated Microbes: An Overview

**DOI:** 10.3389/fpls.2018.00452

**Published:** 2018-04-06

**Authors:** Shalini Tiwari, Charu Lata

**Affiliations:** CSIR-National Botanical Research Institute, Lucknow, India

**Keywords:** bioavailability, heavy metals, microbes, remediation, stress, tolerance

## Abstract

Several anthropogenic activities including mining, modern agricultural practices, and industrialization have long-term detrimental effect on our environment. All these factors lead to increase in heavy metal concentration in soil, water, and air. Soil contamination with heavy metals cause several environmental problems and imparts toxic effect on plant as well as animals. In response to these adverse conditions, plants evolve complex molecular and physiological mechanisms for better adaptability, tolerance, and survival. Nowadays conventional breeding and transgenic technology are being used for development of metal stress resistant varieties which, however, are time consuming and labor intensive. Interestingly the use of microbes as an alternate technology for improving metal tolerance of plants is gaining momentum recently. The use of these beneficial microorganisms is considered as one of the most promising methods for safe crop-management practices. Interaction of plants with soil microorganisms can play a vital role in acclimatizing plants to metalliferous environments, and can thus be explored to improve microbe-assisted metal tolerance. Plant-associated microbes decrease metal accumulation in plant tissues and also help to reduce metal bioavailability in soil through various mechanisms. Nowadays, a novel phytobacterial strategy, i.e., genetically transformed bacteria has been used to increase remediation of heavy metals and stress tolerance in plants. This review takes into account our current state of knowledge of the harmful effects of heavy metal stress, the signaling responses to metal stress, and the role of plant-associated microbes in metal stress tolerance. The review also highlights the challenges and opportunities in this continued area of research on plant–microbe–metal interaction.

## Introduction

Heavy metal stress has become a major concern in various terrestrial ecosystems worldwide. Nowadays extensive industrialization imparts detrimental effects on soil as well as on crop productivity by accumulating heavy metals (Shahid et al., 2015). Damage to soil texture, i.e., pH of soil, presence of different elements, and accumulation of heavy metals cause direct and/or indirect reduction of plant growth by adversely affecting various physiological and molecular activities of plants (Panuccio et al., 2009; Hassan et al., 2017). Heavy metals such as Zn, Cu, Mo, Mn, Co, and Ni are essential for crucial biological processes and developmental pathways (Salla et al., 2011; Shahid et al., 2015). However, these metals along with four other highly toxic heavy metals including, arsenic (As), lead (Pb), cadmium (Cd), mercury (Hg), Cr, Al, and Be can reduce crop productivity to a great extent when their concentration rises beyond supraoptimal values (Xiong et al., 2014; Pierart et al., 2015). These toxic elements cause morphological abnormalities, and metabolic disorders that lead to yield reduction in plants (Amari et al., 2017). These abnormalities also give rise to the production of reactive oxygen species (ROS), e.g., superoxide anion radical (O^2-^), H_2_O_2_, and hydroxyl radical (OH^-^), resulting in disruption of the redox homeostasis of cells (Gill and Tuteja, 2010; Pourrut et al., 2011; Ibrahim et al., 2015; Shahid et al., 2015). This redox status misbalance is known to be a major cause of heavy metal toxicity in plants. Earlier studies reported the negative impact of accumulation of heavy metals in food crops on human health (Nabulo et al., 2011; Uzu et al., 2011; Shahid et al., 2015). To withstand heavy metal stress and metal toxicity, plants have evolved numerous defense mechanisms viz reduced heavy metal uptake, sequestration of metal into vacuoles, binding to phytochelatins/metallothioneins, and activation of various antioxidants (Shahid et al., 2015).

To decipher regulatory networks involved in response to heavy metal tolerance in plants, various omics approaches such as transcriptomics, proteomics, and metabolomics are being routinely used (Singh et al., 2016). In combination with different functional genomic approaches, the abovementioned omics approaches help to develop improved varieties with enhanced abiotic stress tolerance (Mosa et al., 2017). Several quantitative trait loci (QTLs) and candidate genes have been identified for zinc, iron, and Cd tolerance in various plant species which can be utilized for crop improvement by marker-assisted selection or QTL pyramiding (Courbot et al., 2007; Meyer et al., 2016; Zhang et al., 2017). Several previous studies have also reported improvement in heavy metal stress tolerance of plants by genetic engineering (Eapen and D’Souza, 2005; Farinati et al., 2010; Verma P.K. et al., 2016; Verma et al., 2017). Further, since plant breeding and genetic engineering is a labor intensive and time consuming process, there is a need to develop newer strategies or techniques that would be helpful for sustained crop production and productivity under heavy metal stress. Plant-associated microbes could be used as an alternate strategy for sustainable agricultural production. Numerous plant-associated microbes namely, bacteria and fungi are known to exhibit plant-growth promoting traits under heavy metal stress. These microbes impart favorable effects on plants via several direct and indirect mechanisms such as biofilm formation, siderophores, exopolysaccharide, and phytohormones production (Tiwari et al., 2016, 2017b). Since microbial heavy metal remediation does not involve any transgenic modifications, it is ethically and societally acceptable. Even though heavy metal tolerance in plants through microbial remediation has been investigated for many years, there is still considerable interest in extensive studies on plant–microbe–metal association due to their direct effects on enhanced biomass production and heavy metal tolerance (Glick, 2003; Taj and Rajkumar, 2016; Hansda and Kumar, 2017). This review thus summarizes the recent advances in plant-associated microbes in metal remediation and stress tolerance in plants.

## Plant Growth Under the Influence of Highly Toxic Metals

Among numerous heavy metals, four heavy metals As, Pb, Cd, and Hg are considered as the most toxic metals by the Agency for Toxic Substances and Disease Registry (ATSDR, 2003), based on their toxicity, frequency of occurrence, and most importantly, their exposure potential to flora and fauna. Origin and impact of these four heavy metals on environment and plant growth are briefly described below.

### Arsenic

Arsenic is a naturally occurring metal which pose serious health hazards to millions of people across the globe (Kumar et al., 2015). It is usually originated via volcanic action, erosion of rocks, and by human activities such as applications of pesticides and wood preservatives, mining and smelting operations (Wang and Mulligan, 2006; Tripathi et al., 2007; Neumann et al., 2010). The contamination of As in groundwater used for irrigation and drinking is a worldwide problem as it not only affects crop productivity, but also accumulates in different plant tissues including grains and contaminates food chain (Verma P.K. et al., 2016). Recently, several studies have been carried out to investigate the physiological and molecular mechanisms of As toxicity, accumulation, detoxification, and tolerance in various plants including rice, lettuce, spinach, and carrot (Kumar et al., 2015). Inorganic arsenate As(V) and arsenite As(III) are two forms of As that exist in the environment. Both As(III) and As(V) are toxic and are regarded as major environmental pollutants based on United States Environmental Protection Agency (USEPA) evaluation (Tripathi et al., 2007; Verma et al., 2017). As(III) is more toxic than As(V) and act by interrupting biological functions in plants via different manner as, for example, it binds to proteins with sulfhydryl groups, interfering with their functions (Verma S. et al., 2016). It also generates ROS, inhibits respiration by binding to vicinal thiols in pyruvate dehydrogenase and 2-oxo-glutarate dehydrogenase, and act indirectly as a mutagen by inducing intrachromosomal homologous recombination (Helleday et al., 2000). On the other hand, in plants, As(V) interferes with oxidative phosphorylation and ATP synthesis during energy metabolism (Carbonell et al., 1998; Verma S. et al., 2016).

### Lead

Lead is one of the most widely and evenly distributed trace metals that exist in various forms in the natural sources. It can affect soil, flora, and fauna health by contaminations from leaded fuels, dust, old lead plumbing pipes, various industrial sites, or even old orchard sites in production where lead arsenate is used (Tangahu et al., 2011). Pb^2+^ is non-biodegradable and its long-term exposure is found to be acutely toxic to both plants and animals and has several harmful effects on biological systems including soil properties [e.g., pH, organic carbon, amorphous iron, and aluminum oxides (FEAL), and cation exchange capacity; Bradham et al., 2006; Pehlivan et al., 2009]. If proper remedial action not taken, high soil Pb levels may never return to normal (Traunfeld and Clement, 2001). Pb impairs various biological processes in plants including seed germination, seedling development, root elongation, transpiration, chlorophyll biosynthesis, and cell division (Pourrut et al., 2011; Kumar et al., 2017). It also changes cell membrane permeability by reacting with active groups of different metabolic enzymes, with the phosphate groups of ADP or ATP, and by replacing essential ions, thus causing phytotoxicity (Pourrut et al., 2011; Kumar et al., 2017). Pb toxicity leads to inhibition of ATP production, induces lipid peroxidation, and DNA damage by over production of ROS.

### Cadmium

Cadmium is considered to be one of the most phytotoxic heavy metals. Since it is highly soluble in water, it is easily taken up by plants representing the main entry pathway into the food chain causing serious human health hazards (Buchet et al., 1990). Cd has been classified as a potent human carcinogen by The International Agency for Research on Cancer (IARC, 1993; Gianazza et al., 2007; Gill and Tuteja, 2011). Interestingly, it has reported that it is commonly released into the arable soil from industrial processes and farming practices (Wagner, 1993) and also that crops are the main source of Cd intake by humans (Satarug et al., 2002; Gill and Tuteja, 2011). Even at low concentrations Cd can severely alter several enzyme activities including those involved in the Calvin cycle, carbohydrate and phosphorus metabolism, and CO_2_ fixation (Sandalio et al., 2001; Verma and Dubey, 2001; Sharma and Dubey, 2006; Gill and Tuteja, 2011) ultimately resulting in stunted growth, chlorosis, leaf epinasty, alterations in chloroplast ultrastructure, inhibition of photosynthesis and pollen germination and tube growth, induction of lipid peroxidation, and alterations in nitrogen (N) and sulfur (S) metabolism and disruption of antioxidant machinery (Gill and Tuteja, 2011).

### Mercury

Mercury is a natural component of the Earth’s crust that accumulates in land and water ecosystems, mainly as a consequence of different anthropological actions such as mining and industrial activities (Järup, 2003; Montero-Palmero et al., 2014). The large input of Hg into the arable lands has resulted in the widespread occurrence of Hg-contamination in the entire food chain. In the environment several forms of Hg exist such as elemental (Hg^0^), inorganic (Hg^2+^), associated with ions (HgS, ClHg_2_, Hg_2_Cl_2_), and organic (CH_3_-Hg) but in agricultural soils the ionic form is predominant (Hg^2+^) (Zhou et al., 2008; Azevedo and Rodriguez, 2012). Increasing evidence has shown that Hg^2+^ can readily accumulate in higher plants (Israr et al., 2006; Yadav, 2010). At lower concentrations Hg^2+^ may not significantly affect plant growth but at higher concentrations it becomes highly phytotoxic to plant cells and can cause visible injuries and physiological disorders (Ortega-Villasante et al., 2005; Zhou et al., 2007). Binding of Hg^2+^ to water channel proteins leads to leaf stomata closure and physical impediment of water flow in plants (Zhang and Tyerman, 1999; Zhou et al., 2008). Additionally, it has also been reported to interfere with mitochondrial activity (Zhou et al., 2008). Mercuric ions are further reported to induce oxidative stress by stimulating generation of ROS in plants leading to disruption of biomembrane lipids and cellular metabolism, as well as increased activities of antioxidant enzymes like SOD, POD, or APX indicating the degree of stress (Cargnelutti et al., 2006; Zhou et al., 2007).

## Heavy Metal Signaling and Tolerance in Plants

**FIGURE 1:**
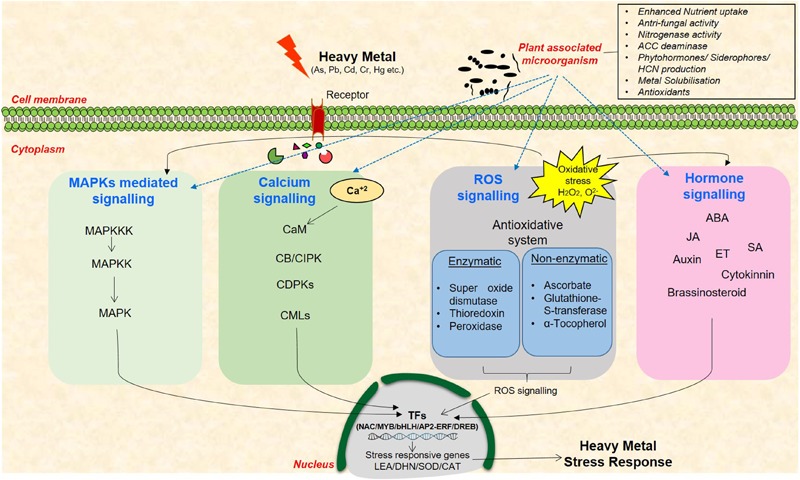
A schematic representation of heavy metal stress signaling cascade in plants and the existing cross-talk among the networks of plant–microbe–metal interaction. These signaling pathways include MAPKs, calcium, ROS, and hormone signaling molecules that mediate signal transduction to enhance the expression of stress-responsive genes.

In the last few decades, the research areas pertaining to plant responses and tolerance to heavy metal stress have rapidly progressed. Several genes that are induced under metal stress have been identified through various omics approaches as, for example, transcriptome analysis in different plants including *Arabidopsis, Brassica*, and *Lycopersicum* revealed role of several transcription factors (TFs) such as *bHLH, bZIP, AP2/ERF*, and *DREB* under heavy metal stress (LeDuc et al., 2006; Shameer et al., 2009; Singh et al., 2016). Use of various proteomics techniques such as 2-D electrophoresis, MALDI-TOF, LC-MS have led to the discovery target proteins that take part in heavy metal detoxification in several plants including *Oryza sativa, Zea mays, Arabidopsis*, and *Populus* sp. (Lingua et al., 2012; Wang et al., 2013; Singh et al., 2016). Similarly, various amino acids, amines, organic acids, phenol, glutathione, and α-tocopherol are some metabolites which have been reported to be involved under heavy metal stress tolerance (Collin et al., 2008; Yusuf et al., 2012; Singh et al., 2016). However, the functions of several of them are still not known owing to the complexity in plant responses to these stresses. Heavy metal stress signal transduction is initiated by receptors/ion channels by perception of stress signal(s) and further by non-protein messengers such as cyclic nucleotides, calcium, and hydrogen ions (**Figure 1**). Several kinases and phosphatases relay the stress signals that further leads to gene expression of various TFs and synthesis of metal-detoxifying peptides (Rao et al., 2011; Islam et al., 2015; Kumar and Trivedi, 2016). Heavy metal(s) activates distinct signaling pathways in plants such as calcium-dependent signaling, mitogen-activated protein kinase signaling, ROS signaling, and hormone signaling that enhance the expression of TFs and/or stress-responsive genes (Dubey et al., 2014; Kumar and Trivedi, 2016). Diverse Ca^2+^ sensors such as calmodulins (CaMs), CaM-like proteins, calcineurin B-like proteins (CBLs), and Ca^2+^-dependent protein kinases (CDPKs) exist in plants that sense, decode, and convey the alterations in cytosolic Ca^2+^ concentration for the stress response (Conde et al., 2011; Steinhorst and Kudla, 2014). Transcript profiling of rice roots exposed to long-term and short-term Cr stress suggested the involvement of CDPKs as their activity increased with increasing Cr(VI) concentration (Huang et al., 2014). In foxtail millet, Ca^2+^ activates antioxidant enzymes and provides tolerance against Cr stress (Fang et al., 2014). Similarly, MAPKs signaling cascade phosphorylate numerous TFs such as ABRE, DREB, bZIP, MYB, MYC, NAC, and WRKY thus influencing metal stress response (Lin and Aarts, 2012; Tiwari et al., 2017a). High levels of Cu and Cd are known to activate distinct MAPKs in *Medicago sativa* (Jonak et al., 2004). Similarly, Cd induces *OsMAPK2* and myelin basic protein (*MBP*) *kinase* gene in rice (Yeh et al., 2004). Several studies have also suggested heavy metal-mediated MAPKs activation via ROS generation, accumulation, and alteration in antioxidant system in *Arabidopsis* and rice (Liu et al., 2010; Kumar and Trivedi, 2016). ROS are also known to disrupt various phytohormone signaling pathways including auxin, ethylene, and JA. A recent study demonstrated that JA exposure improved antioxidant response leading to Cd stress tolerance in rice (Singh and Shah, 2014). Comparative transcriptome analysis of As(III)-treated rice seedlings suggested modulation of signal transduction, plant defense, and hormonal signaling processes such as ABA metabolism (Chakrabarty et al., 2009). The above observations clearly suggest that variation in the levels of phytohormones change plant response to metal stress.

Several reports also indicate the role of signaling molecules in providing plant-associated-beneficial microbes-mediated abiotic stress tolerance in plants as, for example, *MAPK5* was found to be differentially expressed in rice roots treated with *Bacillus amyloliquefaciens*, a plant growth promoting rhizobacteria (PGPR) under salt stress indicating the induction of MAPKs signaling in presence of PGPR in plants (Nautiyal et al., 2013). Altered expression of *At3g57530* responsible for calcium- and CaM-dependent protein kinase activity was reported in *Arabidopsis* under *Pseudomonas putida* and *Pseudomonas fluorescens* treatment (Wang et al., 2005; Srivastava et al., 2012). The expression of several downstream stress-responsive TFs such as MYB, NAC, and bZIP were also found to be modulated by PGPR treatment in several plants including rice, chickpea, and *Arabidopsis* (Srivastava et al., 2012; Tiwari et al., 2016, 2017b). Role of phytohormones ABA, SA, JA, and ethylene have also been elucidated in PGPR inoculated plants under stressed conditions (Tiwari et al., 2016, 2017b). The induction of these genes which are central to heavy metal stress signaling, in the presence of plant-associated microbes as well indicate the complex cross-talk between plant, microbes, and heavy metals in stress response and tolerance. Therefore, an understanding of the intricate metal stress signaling pathways and the existing cross-talk among the networks of plant–microbe–metal interaction is extremely important to elucidate the stress-responsive networks in plants.

## Microbial Remediation of Heavy Metals for Plant Growth Promotion

**FIGURE 2:**
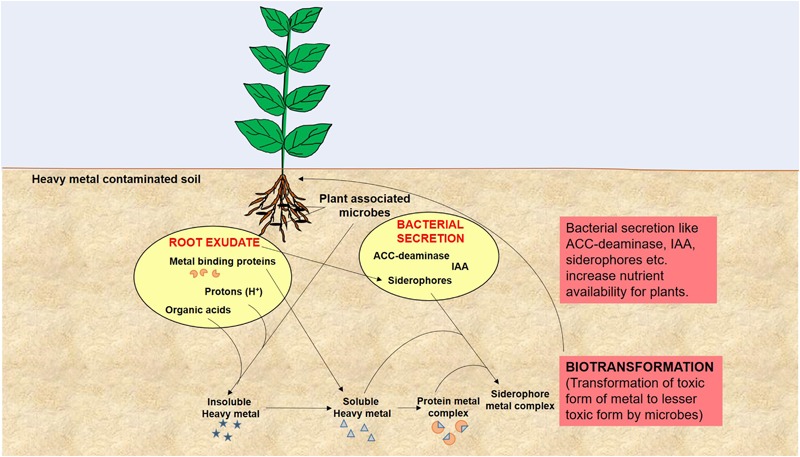
A model of microbial remediation processes involved in heavy metal removal elaborates modulation of plant growth and alteration of soil physicochemical properties by root exudates and bacterial secretion to enhance metal bioavailability and biotransformation that leads to rapid detoxification and/or removal of heavy metal from soil.

Remediation of heavy metals is necessary for the protection and conservation of the environment (Glick, 2010). For the elimination of heavy metals from the environment, numerous physicochemical and biological techniques have been adopted. Physicochemical techniques are rapid but are regarded as challenging due to the cost involved and technical complexity. They also cause adverse effects on soil physical, chemical, and biological properties, and lead to secondary pollution (Glick, 2010; Sheoran et al., 2011; Ali et al., 2013; Ullah et al., 2015). On the other hand, biological remediation is considered as the most effective method of toxic metal removal as these are natural, environment friendly, low cost, and high societal acceptance technologies (Doble and Kumar, 2005). One such technology is the use of plant growth promoting microbes for bioremediation of heavy metal polluted soil and is quite important in the context of global climate change and excessive fertilizer use in agricultural soils (Nautiyal et al., 2013; Tiwari et al., 2016). Microbes are known for enhancement of plant growth and survival under heavy metal stress condition as they have the capability of consuming waste and converting the complex waste into simple non-toxic by products/compounds. This is feasible because microorganisms have developed many resistance mechanisms for survival in the presence of toxic heavy metals in their environment (Thassitou and Arvanitoyannis, 2001; Mustapha and Halimoon, 2015). Microbes also enhance bioavailability of metals from soil by chelation, acidification, and precipitation as, for example, organic acids released by microbes and plant roots lower the soil pH and helps in sequestration of metal ions (Mishra et al., 2017). Microbial remediation processes via plant-associated microbes involved in heavy metal removal is represented in **Figure 2**. These resistance mechanisms developed by microbes include metal sorption, bioaccumulation, and enzymatic oxidation or reduction to a non-toxic form, and efflux of heavy metals from the cell (François et al., 2012; Monteiro et al., 2012; Hrynkiewicz and Baum, 2014; Mustapha and Halimoon, 2015). Here we have provided a list of recently studied plant-associated microbes that respond to various metal stress in plants (**Table 1**).

**Table 1:** List of plant-associated microbes reported for plant growth promotion under heavy metal stress (2010 onward).

S. No.	Microorganisms	Heavy metals	Plants	Reference
1.	*Bacillus cereus, Pseudomonas moraviensis*	Cu, Cr, Co, Cd, Ni, Mn, Pb	*Triticum aestivum*	Hassan et al., 2017
2.	*Microbacterium* sp. CE3R2, *Curtobacterium* sp. NM1R1	Zn, Pb, Cu, As	*Brassica nigra*	Román-Ponce et al., 2017
3.	*Bacteroidetes bacterium, Pseudomonas fluorescens*	Cd, Cu, Pb, Zn	*Brassica napus*	D abrowska et al., 2017
4.	*Kocuria* sp. CRB15	Cu	*Brassica nigra*	Hansda and Kumar, 2017
5.	*Klebsiella pneumoniae*	Cd	*Oryza sativa*	Pramanik et al., 2017
6.	*Enterobacter ludwigii, Klebsiella pneumoniae*	Hg	*Triticum aestivum*	Gontia-Mishra et al., 2016
7.	*Azospirillum*	Pb, Cd	*Panicum virgatum*	Arora et al., 2016
8.	*Enterobacter, Leifsonia, Klebsiella, Bacillus*	Cd	*Zea mays*	Ahmad et al., 2016
9.	*Pseudomonas putida*	Cd	*Eruca sativa*	Kamran et al., 2015
10.	*Rhodococcus erythropolis, Achromobacter* sp., *Microbacterium* sp.	Zn, Cd	*Trifolium repens*	Pereira et al., 2015
11.	*Variovorax paradoxus, Rhodococcus* sp., *Flavobacterium* sp.	Cd	*Brassica juncea*	Belimov et al., 2015
12.	*Bacillus pumilus* E2S2, *Bacillus* sp. E1S2	Cd, Zn	*Sedum plumbizincicola*	Ma et al., 2015
13.	*Rhizobium leguminosarum*	Zn	*Brassica juncea*	Adediran et al., 2015
14.	*Glomus versiforme*	Cd	*Solanum nigrum*	Liu et al., 2015
15.	*Rhizophagus clarus*	Pb	*Cymbopogon citratus*	Lermen et al., 2015
16.	*Pseudomonas* sp. LK9	Cd, Zn, Cu	*Solanum nigrum*	Chen et al., 2014
17.	*Bacillus licheniformis*	Ni	*Oryza sativa*	Jamil et al., 2014
18.	*Rahnella* sp. JN6	Cd, Pb, Zn	*Brassica napus*	He et al., 2013
19.	*Bacillus thuringiensis* GDB-1	As, Cu	*Alnus firma*	Babu et al., 2013
20.	*Ralstonia eutropha, Chryseobacterium humi*	Zn, Cd	*Helianthus annuus*	Marques et al., 2013
21.	*Staphylococcus arlettae*	As	*Brassica juncea*	Srivastava et al., 2013
22.	*Ochrobactrum* sp., *Bacillus* sp.	Cd, Pb, As	*Oryza sativa*	Pandey et al., 2013
23.	*Paenibacillus macerans, Bacillus endophyticus, Bacillus pumilus*	Cu, Ni, Zn	*Brassica juncea*	Tiwari et al., 2012
24.	*Bacillus* sp. MN3-4	Pb	*Alnus firma*	Shin et al., 2012
25.	*Psychrobacter* sp. SRS8	Ni	*Helianthus annuus, Ricinus communis*	Ma et al., 2011
26.	*Bacillus* sp. SLS18	Cd	*Solanum nigrum*	Luo et al., 2011
27.	*Glomus mosseae*	Cd, Pb	*Cajanus cajan*	Garg and Aggarwal, 2011
28.	*Bacillus cereus, Candida parapsilosis*	Fe, Mn, Zn, Cd	*Trifolium repens*	Azcón et al., 2010
29.	*Paecilomyces lilacinus* NH1	Cd	*Solanum nigrum*	Gao et al., 2010
30.	*Bradyrhizobium* sp. 750, *Pseudomonas* sp., *Ochrobactrum cytisi*	Cu, Cd, Pb	*Lupinus luteus*	Dary et al., 2010

### Remediation of Heavy Metals by Bacteria

Bacteria are the most crucial microbial organisms used for the remediation of heavy metal contaminated soils (Chen et al., 2015). Bacteria alleviate heavy metal ion toxicity by immobilizing, mobilizing, uptake, and transformation of heavy metals (Hassan et al., 2017). Moreover, numerous free-living as well as symbiotic PGPR resides in the soil environment around plant root that can positively alter plant growth and its productivity by the production of growth regulators via supplying and facilitating nutrient uptake from soil (Nadeem et al., 2014). Several studies have been reported where PGPR act as potential elicitors for abiotic stress tolerance including heavy metal tolerance (Dary et al., 2010; Tiwari et al., 2016, 2017b). They limit bioavailability of metals by forming complexes with siderophores, particular metabolites, and bacterial transporters (Rajkumar et al., 2010; Ahemad, 2012). These microorganisms of agronomic importance have evolved various mechanisms to avoid heavy metal stress including: (a) transport of metals across cytoplasmic membrane; (b) biosorption and bioaccumulation to the cell walls; (c) metal entrapment in the extracellular capsules; (d) heavy metals precipitation; and (e) metal detoxification via oxidation–reduction (Zubair et al., 2016). Heavy-metal-tolerant PGPR including *Bacillus, Pseudomonas, Streptomyces*, and *Methylobacterium* have the potential to improve growth and production of crops by reducing the detrimental effects of heavy metals (Sessitsch et al., 2013). Previous study reported Cd resistant *Ochrobactrum* sp. and Pb and As resistant *Bacillus* spp. have several PGPR traits that help in bioremediation and growth promotion of a rice cultivar (Pandey et al., 2013). Different rhizobacteria also have been reported that take part in metal accumulation and helps hyperaccumulating plants in uptake of heavy metals and their tolerance (Thijs et al., 2017). Further, it has been reported that use of microbes with some additives for the plants grown in heavy metal polluted soil are more beneficial than without additives (Mishra et al., 2017). A recent study showed that addition of thiosulfate with metal-tolerant microbes enhanced mobilization and uptake of As and Hg in *Brassica juncea* and *Lupinus albus* promoting bioavailability and phytoextraction (Franchi et al., 2017). These methods can aid both the biocontrol and bioremediation process simultaneously in polluted soils.

In spite of these practices, nowadays, the use of genetically transformed bacteria in heavy metal bioremediation is gaining great consideration; however, this limited to laboratory trials only (Gupta and Singh, 2017). Symbiotic relationship between plants and genetically transformed bacteria helps in *in situ* bioremediation of organic pollutants (Ullah et al., 2015; Ashraf et al., 2017). However, only a few evidences are available that highlights the remediation of heavy metals through such symbiotic associations (Ullah et al., 2015). Examples of few genetically engineered PGPR are listed in **Table 2**. Recently elimination of toxic metals through a novel phytobacterial strategy, i.e., via use of genetically transformed PGPR has been suggested (Ullah et al., 2015). Genetically transformed bacteria possess one or more genes to increase remediation of heavy metals. In this context, genes for metal chelators, metal homeostasis, transporters, biodegradative enzymes, metal uptake regulators, and biotic and abiotic stress tolerance are important candidates for making recombinant bacteria (Singh et al., 2011).

**Table 2:** List of genetically modified plant-associated microbes for heavy metal stress tolerance (based on Ullah et al., 2015).

S. No.	Genetically engineered microbe	Modified gene expression	Associated plant	Heavy metal(s)	Reference
1.	*Pseudomonas putida*	Phytochelatin synthase	*Triticum aestivum*	Cd, Hg, Ag	Yong et al., 2014
2.	*Mesorhizobium huakuii*	Metallothionein, phytochelatin synthase	*Astragalus sinicus*	Cd, Cu, Zn, As	Ike et al., 2008
3.	*Mesorhizobium huakuii*	Metallothionein, phytochelatin synthase	*Astragalus sinicus*	Cd	Ike et al., 2007
4.	*Pseudomonas putida*	Expression of metal binding peptide	*Helianthus annuus*	Cd	Wu et al., 2006
5.	*Mesorhizobium huakuii*	Phytochelatin synthase	*Astragalus sinicus*	Cd	Sriprang et al., 2003
6.	*Meshorhizobium huakuii*	Metallothionein	*Astragalus sinicus*	Cd	Sriprang et al., 2002
7.	*Enterobacter cloacae*	EC 4.1.99.4	*Brassica napus*	As	Nie et al., 2002
8.	*Ralstonia eutropha*	Metallothionein	*Nicotiana benthamiana*	Cd	Valls et al., 2000

### Remediation of Heavy Metals by Fungi

Numerous filamentous fungi belonging to the genera *Trichoderma, Penicillium, Aspergillus*, and *Mucor* have been described as having the ability to tolerate heavy metal stress (Ezzouhri et al., 2009; Oladipo et al., 2017). Fungal cell walls have excellent metal binding properties due to presence of negative charge on the different functional groups, e.g., carboxylic, amine or sulfhydryl, phosphate, in different wall components (Tobin, 2001; Ong et al., 2017). A study showed interaction of *Aspergillus niger* var. *tubingensis* Ed8 with Cr(VI) mainly in a reduction process and also in a sorption process (Coreño-Alonso et al., 2014). Previous studies reported reduction in As induced stress in chickpea through *Trichoderma* sp. (Tripathi et al., 2013; Tripathi et al., 2017).

Arbuscular mycorrhizal fungi (AMF) are also one of the most prominent soil microorganisms. They establish direct physical link between soil and plant roots which increase root surface area facilitating nutrient absorption by the plants (Saxena et al., 2017). AM fungi are also involved in alleviating metal toxicity to the host plant (Leyval et al., 1997; Meharg, 2003). The specific role of arbuscular mycorrhizae in the host plant on exposure to heavy metal depends on a variety of factors, including the plant species and ecotype, the fungal species and ecotype, the metal and its availability; soil edaphic conditions, including soil fertility; and plant growth conditions, such as light intensity or root density (Pawlowska and Charvat, 2004). Similar to PGPR, several mechanisms have been hypothesized for toxic metal direction and allocation in plant roots in the presence of AMF including (a) heavy metals bound to cell wall and deposit in the vacuoles of AMF, (b) metal sequestration by the help of siderophores in the soil or into root apoplasm, (c) metals bound to metallothioneins or phytochelatins inside the fungal or plant cells, and (d) metal transporters at the tonoplast of both plants and fungi catalyze the transport of metals from cytoplasm (Jan and Parray, 2016).

## Conclusion and Future Perspectives

Heavy metal contamination and remediation has received considerable attention in today’s world owing to the fact that several heavy metals cannot be degraded and hence persist in the soil. Several strategies have been successfully applied to generate plants which are able to grow in metal contaminated soils and accumulate or tolerate metal stress. Use of microbial approach for heavy metal tolerance and remediation is an eco-friendly and economic approach. Since the plant heavy metal uptake and tolerance depend on various factors, interactions between plant and microbes can play an important role in successful survival and growth of plants in contaminated soils. Plant growth promoting microbes also assist plant growth by changing bioavailability of heavy metal. These beneficial effects exhibited by microbes, together with the suggested interrelationship between heavy metal tolerance and plant growth promoting ability, indicates that their exploitation in remediating metal contaminated soils might have significant potential in near future. In spite of these practices, genetically engineered microbes also have been used for remediation processes. Undoubtedly these engineered microbes have greater remediation potential but their impact on ecosystems needs to be elucidated before commercialization. Despite several findings to date, various steps of regulatory networks via plant-associated microbes in heavy metal stress are still unknown, and more investigations need to be done for unraveling the cross-talk among soil-microbe and metal interaction in different crops. Additionally, synergistic action of plant and microbe and their mechanism for metal mobilization, transformation, and detoxification should also be studied. Further monitoring and managing microbial heavy metal remediation requires the characterization of the fate and behavior of the compounds of interest in the environment. However, at present, it is difficult to understand the environmental impacts of various metals mostly as a consequence of insufficient information being available about them. Thus this highlights the importance of a consistent link between research and development for the assessment and treatment of emerging metal pollutants and the tools, equipment and knowhow that contributes toward the fulfillment of these challenges.

## Author Contributions

ST and CL wrote and reviewed the manuscript.

## Conflict of Interest Statement

The authors declare that the research was conducted in the absence of any commercial or financial relationships that could be construed as a potential conflict of interest.
